# Chilling acclimation provides immunity to stress by altering regulatory networks and inducing genes with protective functions in Cassava

**DOI:** 10.1186/s12870-014-0207-5

**Published:** 2014-08-05

**Authors:** Changying Zeng, Zheng Chen, Jing Xia, Kevin Zhang, Xin Chen, Yufei Zhou, Weiping Bo, Shun Song, Deli Deng, Xin Guo, Bin Wang, Junfei Zhou, Hai Peng, Wenquan Wang, Ming Peng, Weixiong Zhang

**Affiliations:** 1 The Institute of Tropical Bioscience and Biotechnology, Chinese Academy of Tropical Agricultural Sciences, Haikou, China; 2 Institute for Systems Biology, Jianghan University, Wuhan, 430056, Hubei, China; 3 Department of Computer Science and Engineering, Washington University in St. Louis, St. Louis, 63130, MO, USA; 4 Department of Genetics, Washington University School of Medicine, St. Louis, 63110, MO, USA

**Keywords:** Chilling acclimation, Chilling shock, Gene regulation, Cassava, Castor bean

## Abstract

**Background:**

Stress acclimation is an effective mechanism that plants acquired for adaption to dynamic environment. Even though generally considered to be sensitive to low temperature, Cassava, a major tropical crop, can be tolerant to much lower temperature after chilling acclimation. Improvement to chilling resistance could be beneficial to breeding. However, the underlying mechanism and the effects of chilling acclimation on chilling tolerance remain largely unexplored.

**Results:**

In order to understand the mechanism of chilling acclimation, we profiled and analyzed the transcriptome and microRNAome of Cassava, using high-throughput deep sequencing, across the normal condition, a moderate chilling stress (14°C), a harsh stress (4°C) after chilling acclimation (14°C), and a chilling shock from 24°C to 4°C. The results revealed that moderate stress and chilling shock triggered comparable degrees of transcriptional perturbation, and more importantly, about two thirds of differentially expressed genes reversed their expression from up-regulation to down-regulation or vice versa in response to hash stress after experiencing moderate stress. In addition, microRNAs played important roles in the process of this massive genetic circuitry rewiring. Furthermore, function analysis revealed that chilling acclimation helped the plant develop immunity to further harsh stress by exclusively inducing genes with function for nutrient reservation therefore providing protection, whereas chilling shock induced genes with function for viral reproduction therefore causing damage.

**Conclusions:**

Our study revealed, for the first time, the molecular basis of chilling acclimation, and showed potential regulation role of microRNA in chilling response and acclimation in Euphorbia.

## Background

Plants have developed complex defense systems to cope with and combat against harsh environmental stress. After environmental stress, e.g., chilly temperature, most plants gain or increase stress tolerance, resulting in stress acclimation [1],[2]. Chilling acclimation is a favorable trait that is critical for plant growth, reproduction and survival. It helps defeat various chilling-related catastrophic outcomes, such as growth retardation, chlorosis, necrosis, and yield reduction [3]. Chilling resistance can be further classified into above-zero temperature tolerance for tropical plants and sub-zero temperature tolerance for temperates [4].

Cold stress can be classified as chilling (<20°C) and freezing (<0°C) stress [5]. Tropical plants can be injured by above-zero chilling temperature; chilling-injured leaves may become purple or reddish and in some cases wilt [6]. Chilling responses of tropical plants has been documented: an oxidative signaling regulatory network triggers an early response to chilling stress in Japonica rice [7], and exogenous ABA can induce freezing tolerance in chilling sensitive rice seedlings [8]. Further, global expression profiling of chilling induced genes in the chilling tolerant Japonica rice suggests a role of ABA signaling in chilling tolerance [9]. Fatty acid desaturation of organelle membrane lipids related ACYL-LIPID DESATURASE2 is required for chilling and freezing tolerance in Arabidopsis [10]. A chloroplast-targeted protein complex stabilization related DnaJ protein contributes to maintenance of photosystem II under chilling stress in tomato [11].

Chilling response and chilling acclimation may alter gene regulatory circuitry [12]. However, the scale and mechanism of reprogramming of gene regulation as well as the molecular components involved remain to be investigated in important tropical crops, such as Cassava. Moreover, although small noncoding RNAs (sncRNAs), particularly microRNAs (miRNAs), have been recognized as essential post-transcriptional gene regulators in plant development and stress responses [12]–[14], their functions in chilling acclimation have not yet been well documented.

Most previous studies on chilling stress focus on model plants, typically *Arabidopsis* and rice, whereas little has been done on Euphorbia, a genus of tropical plants. Many Euphorbiaceous plants, e.g., Cassava and castor bean, are agri-economically important. Cassava (*Manihot esculenta*) is a major source of carbohydrates for over 500 million people in the developing countries in the tropics and sub-tropics [15]. It is also a major source of industrial material, for biofuel production for example [15],[16]. Cassava is remarkably tolerant to drought and low-fertility soils. However, it is sensitive to low temperature, and chilling injury often occurs in spring planting and autumn harvest seasons.

In this genome-wide study we analyzed transcriptome variations of Cassava plants in response to chilling and during chilling acclimation, aiming at elucidating gene regulatory networks underlying chilling acclimation. Specifically we compared the gene expression variations in responses to dramatic temperature decreases and during chilling acclimation in reference to the normal growth condition. We profiled the expression of protein-coding genes and sncRNA species using Next Generation (NextGen) sequencing. By analyzing more than 35.3 million sequencing reads from 4 mRNA libraries and 25.6 million reads from 4 sncRNA libraries, we identified differentially expressed mRNA and miRNA genes, from which we further identified and analyzed mRNA and miRNA genes that are critical to chilling acclimation.

## Results

### Exploring chilling response and acclimation in Cassava

We profiled the transcriptome and microRNAome of SC124, a widely planted Cassava cultivar in China. SC124 is sensitive to chilling and can be exploited to study chilling response and acclimation. The profiling experiments were carried out under three chilling stress treatments (detailed in Methods and illustrated in Additional file 1: Figure S1): 1) gradual *chilling acclimation* (CA) where plants grown in the normal condition of 24°C were stressed to 14°C; 2) *chilling* stress after *chilling acclimation* (CCA) where plants after 5 days of the CA treatment were transferred further from 14°C to 4°C; and 3) *chilling shock* (CS) where plants were experienced a dramatic temperature drop from 24°C to 4°C (see Methods for detail). For comparison, plants grown continuously under 24°C were used as the *normal control* (NC). Total RNA was extracted from three organs/tissues of the plants at the 6 h, 24 h and 5d of the corresponding stress treatments and the normal control in order to account for initial and secondary responses as well as functional adaption to chilling stresses.

Distinct symptoms of chilling stress of CS and CCA were observed at the end of these stress experiments (Additional file 1: Figure S1). The CCA treated plants were more chilling resistant than the CS treated plants at 4°C: fewer leaves wilted and more leaves stayed upright. Four physiological traits were measured to further evaluate the impact of chilling stress (Additional file 1: Figure S2). While there was no statistically significant changes in leaf falling (Additional file 1: Figure S2A), chlorophyll content only decreased in CS condition (Additional file 1: Figure S2B), malondialdehyde content (Additional file 1: Figure S2C) and leaf proline content (Additional file 1: Figure S2D) increased after one of the three chilling treatments. Malondialdehyde content was progressively elevated with the severity of stress, from CA, CCA to CS. Proline content exhibited different variation patterns. It increased the most under CS and had the least variation under CCA (Additional file 1: Figure S2D).

### Chilling stresses trigger significant transcriptome and microRNAome variations

**Figure 1:**
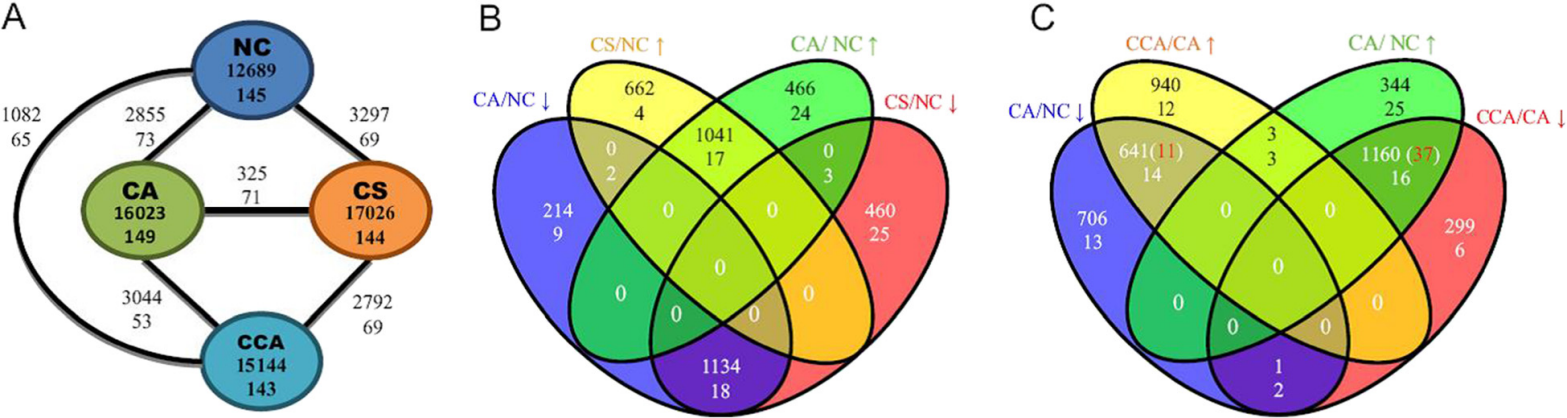
**Results of transcriptome and microRNAome profiling and their variations across the normal condition (NC) and the three chilling stress treatments – chilling shock (CS), chilling acclimation (CA) and chilling after chilling acclimation (CCA). (A)** The numbers of expressed and differentially expressed (DE) genes and miRNAs across the four conditions. The two numbers in an oval are the numbers of expressed genes (the first line) and miRNAs (the second line). The two numbers on an edge are the numbers of DE genes and DE miRNAs between two conditions. **(B)** Relationship between the up- and down-regulated genes and that of miRNAs of CS and CA with respect to NC. The two numbers within a region are the numbers of DE genes (the first line) and miRNAs (the second line). The figure shows a substantial overlapping between the up-regulated genes (and miRNAs) and overlapping between the down-regulated genes (and miRNAs) of CS and CA. **(C)** Similar to **(B)**; relationship between the up- and down-regulated genes and that of miRNAs when going from NC to AC and then from AC to ACC. The figure shows a little overlapping between the up-regulated genes (and miRNAs) and overlapping between the down-regulated genes (and miRNAs) in the comparison.

In order to appreciate the impact of chilling stress, we profiled the expression of mRNA genes and small-noncoding RNA (sncRNA) species of SC124 after the CA, CCA and CS treatments as well as under the normal condition (NC) using Illumina GAIIx (see Methods, sequencing data in NCBI/GEO, accession # GSE52178). Overall ~33 million (>90%) of raw sequencing reads from mRNA genes, or RNA-seq reads, were high-quality (or qualified) reads, among which more than 80% could be mapped to the Cassava reference genome (http://phytozome.net) allowing one mismatch (Additional file 1: Table S1). With the criterion of at least 10 reads per million (RPM, see Methods), the mapped reads attributed to 12,689 (37.16% of the 34,151 annotated Cassava mRNA genes), 16,023 (46.92%), 15,144 (44.34%) and 17,026 (49.85%) mRNA genes expressed under the NC, CA, CCA, and CS conditions, respectively (Figure 1A and Additional file 1: Table S1). Comparing the numbers of these expressed genes showed that at least 19% more genes were expressed in any of the three chilling treatments than in the normal condition.

Correspondingly, four small-RNA libraries for the NC, CA, CCA and CS conditions contributed more than 25.6 million raw small-RNA sequencing reads (see Methods, sequencing data in NCBI/GEO, accession # GSE52178), among which 23,468,606 (>91% of the total) were adapter-trimmed, high-quality reads (qualified reads, Additional file 1: Table S2A). Among the qualified reads, which had lengths peaked at 21- and 24-nt, 53.40% and 73.18% could be mapped to the Cassava reference genome allowing zero and one mismatch (Additional file 1: Figure S3 and Tables S2B and S2C), respectively. Based on a set of stringent criteria (see Methods), 61 novel miRNAs from 46 miRNA families were identified. In the total of 154 (93 known and 61 novel and newly annotated) miRNAs that were detected, 145, 149, 143 and 144 were expressed in the NC, CA, CCA, and CS conditions, respectively (Figure 1A).

### Severe and moderate stresses had comparable impact on transcriptome and microRNAome

Among the three stress treatments, chilling shock (CS) perturbed the transcriptome the most as it triggered the largest number of genes to express and had the largest number of differentially expressed (DE) genes, detected by two stringent criteria (see Methods), with respect to the normal condition (NC) (Additional file 1: Table S1 and Figure 1A). The mild chilling stress, chilling acclimation (CA), came in the second.

Although CA and CS reached very different temperature – the former at 14°C whereas the latter at 4°C – the amount and extent of transcriptome variations that they had induced seemed to be comparable in terms of both expressed and differentially expressed genes. Furthermore, there was only a strikingly small difference between the perturbed transcriptomes they induced. Among the 2,855 and 3,297 DE genes of CA and CS in reference to NC, respectively, 2,175 (1,134 plus 1,041 in Figure 1B, 76.2% in CA and 66.0% in CS) were common. More specifically, among these DE genes, 62.72% (1,134 out of 1,808) were down-regulated and 47.99% (1,041 out of 2,169) were up-regulated along the same direction in CA vs NC and CS vs NC. Nonetheless, none of these DE genes were regulated in the opposite directions under the mild and severe chilling treatments (Figure 1B). In concordance, only 325 genes were DE between the CA and CS treatments (Figure 1A), further reflected by the similar expression patterns of the DE genes in CA and CS (Additional file 1: Figure S4). Furthermore, the microRNAome variations caused by CA and CS, reflected by the DE miRNAs, were in concordance with that of transcriptome variations. A large portion of DE miRNAs of CA and CS were in common; in particular, 34.0% (17 out of 50) up-regulated miRNAs were shared by CA and CS, and 31.5% (18 out of 57) down-regulated miRNAs were common to CA and CS. It is important to note that neither DE mRNA genes nor DE miRNAs reversed their expression from up-regulation to down-regulation or vice versa between the CS and CA treatments (Figure 1B).

These results on transcriptome variation were confirmed by a functional enrichment analysis; the two chilling treatments perturbed biological processes of similar functions (Additional file 2: Table S3A). Most of the enriched biological processes of the DE genes of CS and CA with respect to NC were in common, which included translation (GO:0006412, FDR < 1.16x10^−06^), photosynthesis (GO:0015979, FDR < 0.0162), L-serine metabolic process (GO:0006563, FDR < 0.0517) and various other metabolic processes. It is not surprising to observe that the two sets of DE genes share common enriched biological processes, because more than half of these genes were in common. Indeed, translation (FDR < 1.97x10^−08^), photosynthesis (FDR < 0.0244), and L-serine metabolic process (FDR < 0.0244) are also enriched in these 2,175 common DE genes (Additional file 2: Table S3B). Nevertheless, the stress-specific DE genes, i.e., DE genes that were specific to CA vs NC (680 out of 2,855) and that specific to CS vs NC (1,122 out of 3,297), were also enriched with the same biological processes as the commonly shared DE genes. For example, translation (FDR < 3.84x10^−05^) was enriched in the DE genes specific to CA vs NC and photosynthesis (FDR < 3.72x10^−09^) was enriched in the DE genes specific to CS vs NC (Additional file 2: Table S3B). In short, these results suggested that CA and CS perturbed similar biological processes.

### Chilling after chilling acclimation reversed the expression of a large portion of DE genes

In stark contrast, chilling stress after chilling acclimation (CCA) altered the transcriptome the least among the three stress treatments despite that it ultimately reached 4°C as CS did. Surprisingly, the number of DE genes of CCA with respect to NC was approximately one third of that of CS or CA (Figure 1A and B). Further, 2,792 genes were DE between CCA and CS and 3,044 genes were DE between CCA and CA, which are eight times more than the 325 DE genes between CS and CA (Figure 1A).

In order to appreciate the role that chilling acclimation plays in stress response, we compared the DE genes and DE miRNAs of CA and CCA. A total of 2,855 and 1,082 genes were DE in CA and CCA with respect to NC, respectively. This more than 2.6 fold difference between the two sets of DE genes alluded to a genome-wide transcriptome alteration after stress acclimation in that harsh stress after chilling acclimation reversed the transcriptome changes triggered by the moderate chilling stress. Indeed, the expressions of about two thirds of the up- or down-regulated genes in CA with respect to NC were, respectively, reversed to down- or up-regulated in CCA with respect to CA (Figure 1C). Among the 1,507 up-regulated genes after the initial temperature decrease from the normal condition to CA at 14°C, 1,160 (77.0%) changed to down-regulated when going from CA to CCA at 4°C; likewise, among the 1,348 down-regulated genes after going from NC to CA, 641 (47.6%) genes reversed to up-regulated after going from CA to CCA (Figure 1C). In contrast, even though the temperature kept decreasing from CA to CCA, only 1 down-regulated gene was further down-regulated and 3 up-regulated genes were further up-regulated (Figure 1C).

**Figure 2:**
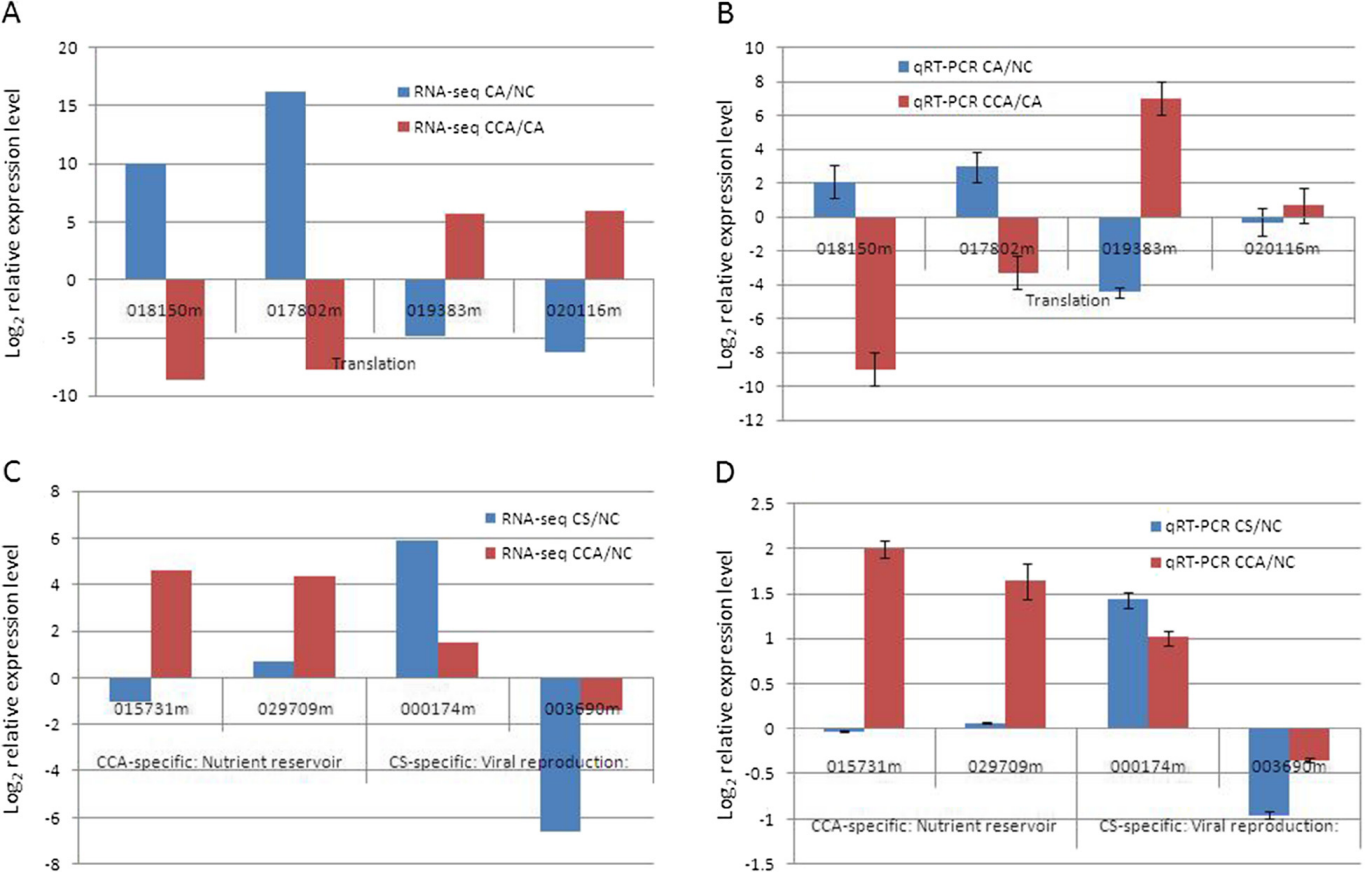
**Experiment validation of reversely expressed genes from CA/NC to CCA/CA and differentially expressed genes specific to CCA or CS.** The reversed expression patterns of four translation-related genes are firstly shown in **(A)** RNA-Seq data and are then confirmed by **(B)** qRT-PCR. The expression patterns of genes, which are associated with nutrient reservoir specific to CCA and which are associated with viral reproduction specific to CS are shown in **(C)** RNA-Seq data and are then confirmed by **(D)** qRT-PCR. We remove the prefix of gene names for simplicity; e.g. “018150 m” is short for “Cassava4.1_018150m”. The expression level of each gene is averaged among three replicates and is normalized by Cassava actin gene in the qRT-PCR assay. The y-axis indicates the relative expression level of a gene in a given condition with respect to the normal control (NC) in log scale. (*Gene list*, 018150 m: ubiquitin 6, 017802 m: ribosomal protein L11 family protein; 019383 m: ribosomal protein L31e family protein; 020116 m: zinc-binding ribosomal protein family protein; 015731 m and 029709 m: RmlC-like cupins superfamily protein; 000174 m: unknown protein; 003690 m: ROP interactive partner 3).

Interestingly, miRNAs might be responsible for the reversion of some of the DE genes to their original expression levels. Specifically, 14 and 16 (87.5%) miRNAs that were down- and up-regulated from NC to CA reversed, respectively, to up- and down-regulation going from CA to CCA; furthermore, 37.0% (30 out of 81) of the DE miRNAs reversed their expression directions (Figure 1C). Importantly, these 30 DE miRNAs targeted 1,198 mRNA genes, among which 48 were DE and reversed their expression directions going from NC to CA and to CCA (Figures 1C and Additional file 1: Figure S5 and Additional file 3: Table S4). This observation suggested that 48 mRNAs with the reversed expression levels might be negatively regulated by the 30 DE miRNAs, which also have reversed expression patterns. The 1,801 (641 plus 1,160) reversely regulated DE genes in the treatments from NC to CA and then from CA to CCA have enriched biological processes such as translation (FDR < 3.69x10-15), superoxide metabolic process (FDR < 0.0052), and mismatch repair (FDR < 0.061) (Additional file 2: Table S3C). We experimentally tested 4 of the reversely regulated genes related to translation (ribosomal protein L11, ubiquitin 6, ribosomal protein L31e, and zinc-binding ribosomal protein) using qRT-PCR (see Methods). The reversed expression patterns of these 4 translation-related genes are consistent with the RNA-seq data and can be confirmed by qRT-PCR assay both under CA/NC and under CCA/CA in the corresponding regulation direction regardless of the magnitude (Figure 2A and B).

### Chilling acclimation prepared the plant to fend off adverse effects of further stress

The significant transcriptome and microRNAome changes, which reversed most DE genes and a substantial number of miRNAs in chilling after chilling acclimation, were in concordance with the mild symptoms of chilling stress of CCA in comparison with the symptom of CS (Additional file 1: Figure S1). This suggested that as the temperature further decreased to 4°C after the initial moderate stress, the plant was able to better adapt to further harsh stress and effectively recover some of the perturbation to biological processes or pathways that had been altered. In other words, stress acclimation (i.e., CA) helped the plant develop a kind of immunity against adverse impact of chilliness at 4°C. Moreover, miRNAs played a role in this process by regulating the expression of some mRNA genes.

**Figure 3:**
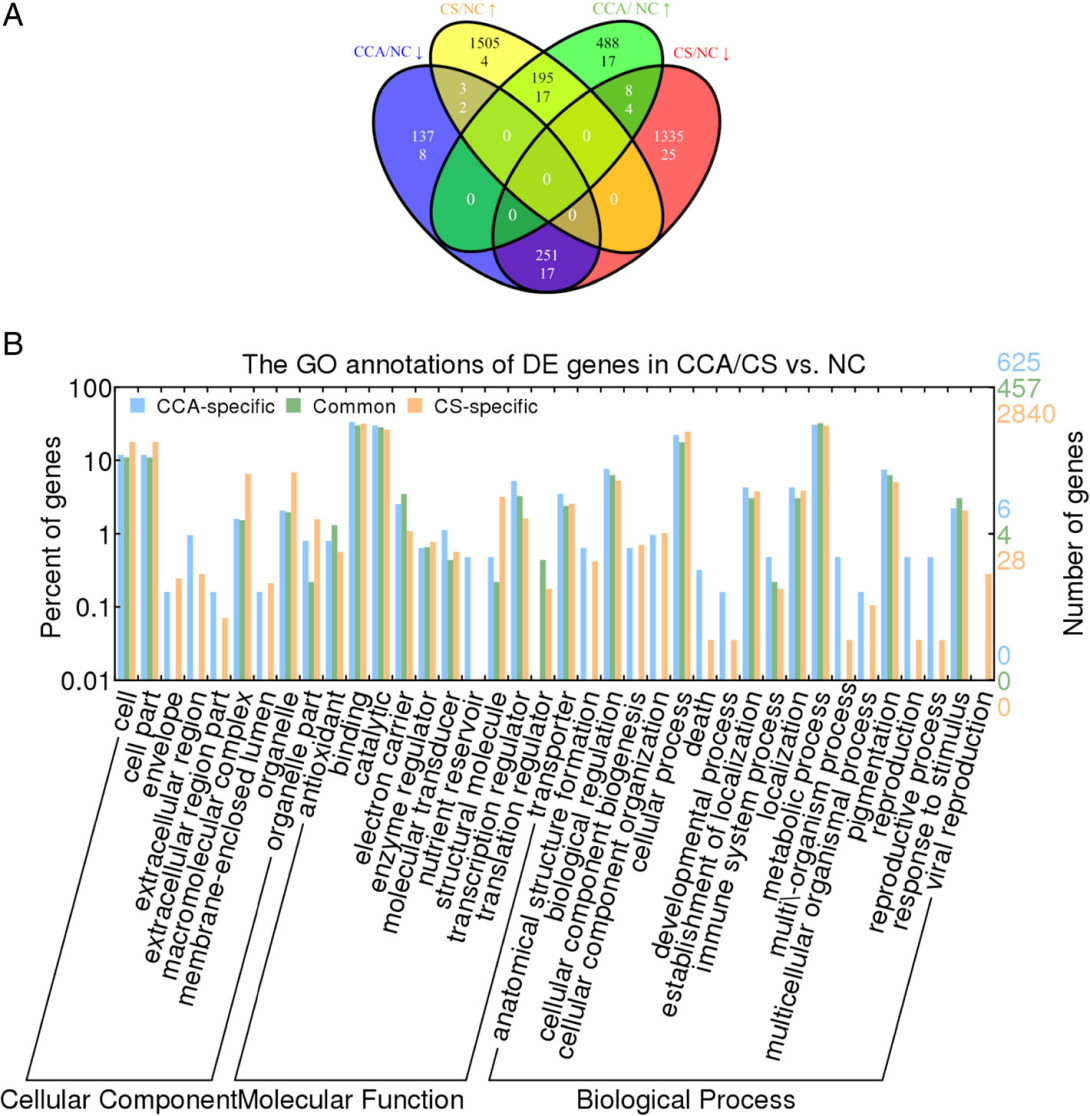
**Common and distinct functions of DE genes in CCA and CS with respect to NC. (A)** Relationship between the up- and down-regulated genes and that of the DE mRNAs in CS and CCA with respect to NC. The two numbers within a region are the numbers of DE genes (the first line) and miRNAs (the second line). **(B)** Biological processes, molecular functions and cellular components that were affected by the DE genes of CCA and CS. The function of nutrient reservoir function is exclusively associated with some of the DE genes of CCA, whereas the process of viral reproduction is exclusively associated with some of the DE genes of CS.

A direct comparison between the transcriptomes of and the biological processes affected by CCA and CS further confirmed our observation. Even though both the CCA and CS treatments reached the same temperature of 4°C, plants that had experienced chilling acclimation at CCA exhibited drastically different transcriptome from plants that had subjected to CS. Similar to the difference between CA and CCA, there was also a huge, more than 3.0 fold, disparity between the number of DE genes of CCA (i.e.,1,082 genes) and that of CS (i.e., 3,297 genes) with respect to NC (Figure 1A). Only 446 of these DE genes were common in both chilling treatments, while 57.8% (625 out of 1,082) of them were specifically DE in CCA vs NC and 86.1% (2,840 out of 3,297) of them were specifically DE in CS vs NC (Figure 3A). This indicated that the degree of transcriptome perturbation due to CCA and CS were substantially different. The functions of the two sets of DE genes for CCA and CS helped reveal the different biological functions of the common and condition-specific genes (Figure 3B). As expected, many biological processes, such as metabolic process and responsive to stimulus, were perturbed under both chilling conditions since 446 DE genes were common to both CCA and CS (Additional file 2: Table S3A and Figure 3A). However, 8 biological processes (i.e. anatomical structure formation, cellular component biogenesis and organization, death, developmental process, multi-organismal process, multicellular organismal process and reproductive process) were not present in the common genes but exclusively appeared in the CCA/CS specific genes. These genes function in 4 different cellular components (envelope, extracellular region, extracellular region part, and membrane-enclosed lumen). The 8 biological processes, which were either associated with the anabolic, growth-promoting state or the catabolic, growth-suppressing state, play a key role in leading Cassava to distinct responses under the CCA and CS treatments. The cellular components of these CCA/CS specific DE genes are closely related to membrane rigidification caused by chilling stress.

In addition, one molecular function was specific to DE genes in CCA and one biological process was specific to CS (Figure 3B). The term nutrient reservoir was only present in DE genes in CCA condition, suggesting their possible protective activities in plants undergone chilling acclimation. Two RmlC-like cupins superfamily protein-coding genes (015731 m and 029709 m) were associated with nutrient reservoir. They were expressed at normal expression levels under the CS condition, but in contrast, they were overexpressed 4 folds under the CCA condition with respect to NC (Figure 2C and D). In contrary, the CS treatment triggered the process of viral reproduction, which did not appear in the CCA treatment. From the RNA-seq data, eight DE genes were associated with viral reproduction, and six out of these eight DE genes were overexpressed in both CA and CS, but expressed normally in CCA. The other two genes were down-regulated by 5–6 folds in CA and CS, but were again expressed normally in CCA. One up-regulated unannotated protein (000174 m) and one down-regulated ROP interactive partner 3 (003690 m) were validated by qRT-PCR methods (Figure 2C and D).

### MicroRNAs contributed to chilling response and stress acclimation

As a major post-transcriptional gene regulator, miRNAs mediate the expression of their target genes in adaptation to environmental stress. To appreciate miRNA functions and gain insight into the complex regulatory networks in chilling response in Cassava, we exploited and combined the large collection of mRNA and small-RNA profiling data for an integrated transcriptome and microRNAome analysis. Based on the small-RNA profiling data, we identified 61 novel miRNAs in Cassava and 121 DE miRNAs in one of the six comparisons we considered (Figure 1A).

**Figure 4:**
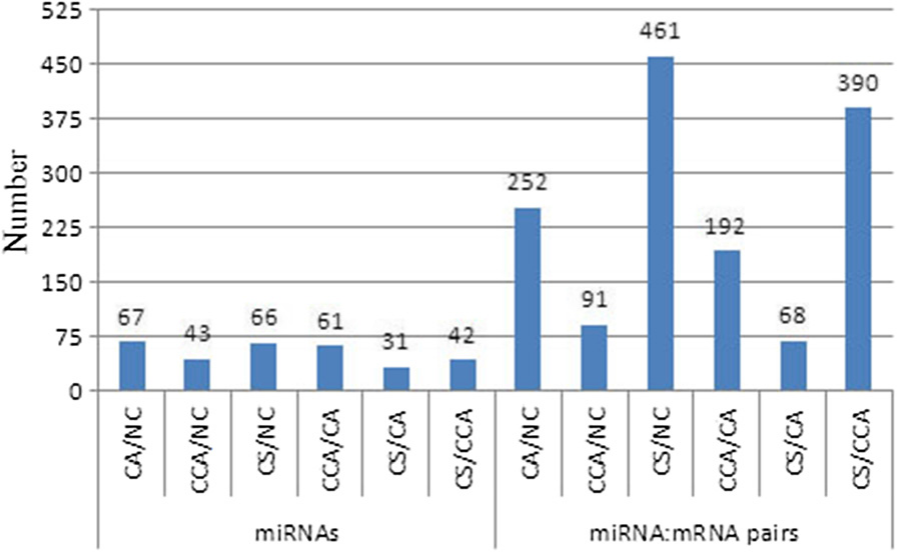
**The DE genes targeted by DE miRNAs among six comparisons.** The number of miRNAs, e.g., listed under “CA/NC”, refers to the number of DE miRNAs in the CA condition with respect to NC. The number of miRNA:mRNA pairs, e.g., listed under “CA/NC”, is the number of mRNA targets that are not only DE but also anti-correlated with their targeting miRNAs in the CA condition with respect to NC.

We identified anti-correlated pairs of DE miRNAs and DE mRNA target genes in 6 comparisons across the stress conditions (Additional file 4: Table S5A). The DE miRNAs played a role in regulating the transcriptome responses to chilling stresses. More than 30 miRNAs regulated at least two potential mRNA targets in each comparison. The regulatory effect of these DE miRNAs was most profound under CS with respect to NC and CCA conditions (Figure 4). We further investigated the miRNA-regulated DE genes that were associated with the significantly enriched pathways in the pairwise comparisons among six conditions (Additional file 4: Table S5B). These DE genes were regulated by miRNAs under at least one of the chilling stresses with respect to NC, while few of them were DE across any of two stress conditions (Additional file 4: Table S5B). Biosynthetic process (29.0%), cellular protein modification process (18.2%), response to stress (11.1%) and metabolic process (10.4%) were the top 4 biological processes that had the largest ratios of genes being regulated by miRNAs to the total number of genes on a given enriched pathway (Additional file 4: Table S5B). As expected, one miRNA can regulate one or several genes, and one mRNA gene may be targeted by multiple miRNAs. For example, 4 genes (005409 m, 006360 m, 006048 m, 005437 m and 005421 m) targeted by miR399 were enriched in the same biosynthetic process in CA vs. NC, at the same time, one gene (012052 m) enriched in oxidation-reduction process and 3 genes (033858 m, 014142 m and 000730 m) enriched in metabolic process under CCA vs. NC were all potentially targeted by miR396a/b/c/d, and novel-3 could target one gene (013577 m) enriched in metabolic process under both CCA vs. NC and CS vs. NC (Additional file 4: Table S5B). Surprisingly, one translation-related gene regulated by miR172 was differentially expressed in four comparisons: CA vs. NC, CS vs. NC, CCA vs. CA and CS vs. CCA, indicating that the interaction between miR172 and 018488 m played regulatory roles in CA and CS, but not in NC and CCA. In short, miRNAs are one of the key regulating factors during the processes of low temperature adaptation in Cassava.

**Figure 5:**
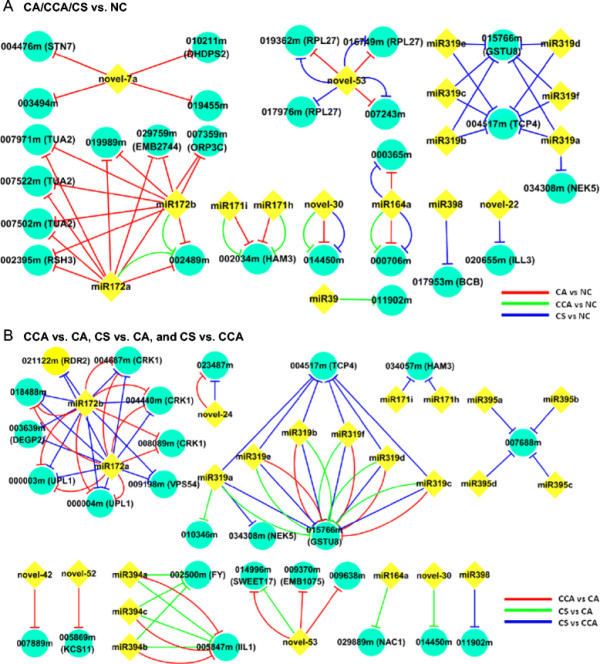
**Regulatory networks showing the relationship between differentially expressed miRNAs and their anti-correlated, differentially expressed target mRNAs.** The yellow diamonds represent miRNAs and the blue circles denote target mRNAs. An edge between a pair of miRNA and mRNA indicates their anti-correlation relationship across two conditions, which is color coded. **(A)** the upper panel is referred to one of three chilling treatment (CA, CCA and CS) with respect to NC, which assigned to red, green and blue line, respectively; **(B)** the bottom panel is referred to in-between comparison of three chilling treatments, including CCA vs. CA (red), CS vs. CA (green) and CS vs. CCA (blue).

The effect of miRNAs on their targets is reflected by the anti-correlated expression patterns between miRNAs and their mRNA targets because the major function of plant miRNAs is mRNA cleavage. We further experimentally tested 17 pairs of anti-correlated miRNAs and target mRNAs, as initially detected by the sequencing data (Figure 5). We first examined cleavage cites of miRNAs on their target genes by 5′RACE (see Methods). The cleavage sites of 13 (76.5%) of the 17 pairs were validated and the cleavage sites were within the regions of miRNA binding sites (Table 1). As expected, many of these anti-correlated miRNAs and mRNAs were related to stress responses, such as novel16-POS, where POS is associated with scavenging hydrogen peroxide, and miR398-EC, where EC is supposed to maintain the membrane potential via electron carrier. Therefore, these identified anti-correlated pairs of miRNA and mRNAs from the profiling data should be valuable candidates and subject to further investigation. NF-YA family, targeted by miR169, has been recently found as an adaptive response to adverse environmental conditions [17]. Note that the cleavage site of NFYA10 by miR169 was indeed detected in our 5’RACE assay, while the anti-correlation between miR169 and NF-YA10 was not detected due to low reads number of miR169 in the RNA-seq data (data not published).

**Table 1:** The cleavage site of anti-correlated miRNA:mRNA pairs detected by 5′RACE assay

**miR-Target**	**ID**	Cleavage sites*			**Anti-correlation**^**§**^						**Annotation**
		**NC**	**CA + CCA**	**CS**	**CA/NC**	**CCA/NC**	**CS/NC**	**CCA/CA**	**CS/CA**	**CS/CCA**	
novel7a-DHDSP2	010211 m	4,±			+						dihydrodipicolinate synthase
Novel53-RPL27	016749 m		21,±	±	+		-				ribosomal protein large subunit 27
miR171hi-HAM3(1)	002034 m		10(5),7(2),-	10,7,3,-	+	+					GRAS family transcription factor
miR395-ATRX	000210 m		9(11)	9,-	+		+				P-loop containing nucleoside triphosphate
											hydrolases superfamily protein
novel16-POS	033835 m		±	5,0,±	+						Peroxidase superfamily protein
Novel42-LMDH	007889 m		-	11,±				-			lactate/malate dehydrogenase family protein
miR394-FY	002500 m		4,3,1(2),-	4,-						+	Transducin/WD40 repeat-like superfamily protein
miR171hi-HAM3(2)	034057 m		10(3),7,-	10(5),7(3),-						+	GRAS family transcription factor
miR319-TCP4	004517 m			8,-			+			+	TCP family transcription factor 4
novel30-CP	014450 m	12,10			+	+	+		-		Cyclase family protein
miR164-NAC	029889 m		10(7)	10(9)		+	+		+		NAC domain containing protein 1
Novel53-SWEET17	014996 m		22,17(2),-	±		+		+	-		Nodulin MtN3 family protein
miR398-EC	011902 m		±	10(2),±		-				+	copper ion binding/electron carrier
miR319-GSTU8	015766 m						-	-	-	-	glutathione S-transferase TAU 8
miR172-TUA	007502 m	-			-						tubulin alpha-2 chain
miR172-LMP	002489 m		+	-	-	-					LMBR1-like membrane protein
miR394-IIL1	005847 m			-	-			-	-		isopropyl malate isomerase large subunit 1

The cleavage sites of 13 (out of 17, 76.5%) miRNA:mRNA pairs were detected within miRNA binding regions. Five (the first five from the top), three (the subsequent three) and five pairs (the next subsequent five) of them were anti-correlated in the three chilling stress conditions with respect to the NC condition, in the other three comparisons and both two comparisons according to RNA-seq data, respectively. The cleavage sites of the remaining four pairs (the last four) failed to be detected in the basepairing regions of corresponding target genes. The “*” column lists number of miRNAs that are complementary with target cleavage sites in binding regions; the “+” and “-” symbols refer to cleavage sites located in upstream and downstream of miRNA:target binding regions, respectively. In the “§” column, “+” means that there is an anti-correlation between a miRNA and its target gene based on sequencing data, and the cleavage site of miRNA:target is located in the basepairing region in 5′RACE assay, and “-” means that there is an anti-correlation in sequencing data, whereas the cleavage site of miRNA:target is not within the basepairing region but either upstream or downstream to the basepairing region.

## Discussion

This is the first genome-wide, systematic study of chilling stress and acclimation in Euphorbia; we integrated genome-scale, sequencing-based profiling data on protein-coding genes and miRNAs on a chilling sensitive cultivar of Cassava, the most important crop in the tropical regions. The study identified critical genes and miRNAs are responsive to chilling stresses and gained insights into the biological processes underlying chilling acclimation. We expect our findings to have a potential impact on plant biology and agriculture. In particular, exposing tropical or subtropical crops to sub-optimal temperatures for stress acclimation can enhance their chilling resistance.

### Comparable effects of severe and moderate stress

Based on a comparative transcriptome analyses of potato (*S. tuberosum*) and *A. thaliana*, Carvallo et al. [18] reported conserved gene expression patterns between the two species during chilling acclimation despite their independent evolution over 100 millions of years. In a large scale evolutionary studies across various species, Preston and Sandve [19] further documented that despite independent development separated by evolution over hundred millions of years, some pathways and mechanisms involved in chilling acclimation are similar between bryophytes, monocots, and eudicots. As for different cultivars of the same species with contrasting responses to chilling stress, their comprehensive comparative studies reveal relatively similar initial transcriptional responses and more diverse downstream molecular changes. While functional categories for genes responding to late phase chilling stress are diverse, ranging from functional adaptation to continuous stress, the early responses have been found to be related to transcription regulation and signal transduction [20]. Similarly, Usadel et al. [21] reported that Arabidopsis has qualitatively similar responses to 17, 14, 12, 10 and 8°C, regardless the degrees of temperature change. We also confirmed this observation in our CA and CS experiments with similar disruptions to the transcriptome. Moreover, we reported here that the chilling-sensitive Cassava could trigger similar transcriptional responses when the Cassava plant was subject to chilling stresses close to freezing temperature (4°C).

### Chilling acclimation helps plants better withstand severe chilling stress

Our results revealed that the largest numbers of expressed and DE genes appeared in the chilling shock (CS) stressed plants. This suggested that a more severe transcriptome perturbation in Cassava plants that had experienced dramatic temperature decreasing (CS) compared to plants that were treated by mild chilling stress (CA) and were stressed at same temperature after acclimation with mild chilling stress (CCA). Surprisingly, more than 65% of the up- or down-regulated genes in CA vs. NC were respectively swapped to down- or up-regulation in CCA vs. CA, indicating a remarkable reversing effect on gene expression and a dramatic rewiring of gene regulatory networks in plants after experiencing chilling acclimation. Such antagonistic regulation patterns and regulatory network changes reflected plants’ profound adaptation to stress through the process of chilling acclimation.

In chilling sensitive plants, a major adjustment is the physical transition of cell membrane from a flexible liquid-crystalline to a solid gel phase, resulting in increased permeability that leads to cellular leakiness and ion imbalance. In Cassava, significant accumulation of hydrogen peroxide (H2O2) have been observed after 4 h chilling exposure; at the same time, the enzyme activities and up-regulation of four ROS-scavenging transcripts, including catalase (CAT), superoxide dismutase (SOD), and glutathione transferase (GST), have been observed and verified [22]. The enzymatic antioxidants, jointly with low molecular-mass antioxidants that were embedded in membrane and polyunsaturated fatty acids, respond to the oxidative stress. The balance of oxidative capacities and scavenging activities of antioxidants is broken as a consequence of abnormal metabolism and injured cells accumulating toxic metabolites and active oxygen species. In our study, oxidoreductase activity was significantly enriched in all of the chilling stresses compared to the control condition, indicating serious oxidative stresses. Furthermore, the DE genes that were specific to CS or CCA significantly varied in the anatomical structure formation and cellular component. In addition to the fact that the chilling injury index (Malondialdehyde content in Additional file 1: Figure S2C) increased most significantly in the CS condition, it is viable to infer that transmembrane damage and membrane permeability have a significant variation between the CCA and CS conditions. In addition, the processes of cell death and development were also significantly distinct between CCA and CS (Figure 3B), indicating that membrane and metabolism adjustment played an important part in the chilling acclimation in Cassava.

We further experimentally validated some of the genes that reversed their expression patterns under further chilling stress after stress acclimation. Ribosomal protein L11 and ubiquitin 6 were up-regulated from the NC to CA condition and subsequently down-regulated from the CA to CCA condition (Figure 2A and B). Both genes related to ubiquitination can reduce protein synthesis for normal growth and provide diversity of small protein nutrient availability for the later chilling adaption [23]. In contrary, ribosomal protein L31e superfamily and zinc-binding ribosomal proteins, which are related to the growth of meristem tissue, were first down-regulated and then up-regulated from the NC to CA and subsequently to the CCA condition. The expression pattern of these genes can help determine whether a cell is in an anabolic, growth-promoting state or a catabolic, growth-suppressing state [24]. Two previous studies also found that low temperature can induce a large number of genes involved in translation, protein synthesis or nucleosome assembly when transferring from normal condition to sub-optimal temperature in *Arabidopsis*[21],[25],[26]. Ribosome biogenesis is a key process for fundamental translation processes. Perturbations to the dosage of the ribosomal protein subunits regulate overall protein synthesis related biological process [24]. The hydrophilic residues of ribosomal protein L11 can interplay with p53-MDM2 function complex [27],[28] to stabilize and activate ribosomal protein-Mdm2-p53 signaling pathway to response DNA damage and ribosomal stress [23],[29]. Those genes altering the accomplishment of normal translation initiation [30],[31], elongation [32], termination [33], and probably ribosome-recycling [34], are supposed to reduce the rate of protein synthesis. Subsequently, Carroll [35] and Gerashchenko [36] reported that translational proteins have exquisitely sensitive and responsive to environmental fluctuations. Furthermore, Ferreyra [37] argued that this widespread and unequivalent translational component reprogramming can have a “turnover” effect on related mRNAs while inducing the translation of specific mRNAs in adaptation to environmental stress.

In addition to translation reprograming, plant resistance to low temperature depends on responsive speed and scope of transcripts and metabolites involved in cryoprotection and stress responses. We identified two RmlC-like cupins superfamily genes specifically DE in the CCA condition with respect to NC. The RmlC-like cupins superfamily proteins have known functions in biology processes of small nutrient molecule, such as sulfur amino acid, polysaccharide metabolic process, amine and carbohydrate biosynthetic process. These metabolites have known oxidoreductase and disomerase activities [38] and cation and hormone binding capacities [39]. They potentially help stressed plants to maintain normal osmotic potential and to defend water loss.

ROP interactive partner 3 (003690 m) was shown to significantly express in the CS condition, ROP functions as molecular switches in auxin signaling at the cell membrane to regulate many cellular and developmental processes and environmental responses [40]. The balance between the activation and inactivation of ROP helps maintain a non-toxic level of H2O2 to avoid lethal effects [41]. Yun et al. [7] report that early response triggered by oxidative signals is critical for prolonged survival under sub-optimal temperature and that many of the oxidative-mediated changes in gene expression occur within 24 hours of stress. Their transcriptome variations have striking similarities to the transcriptome variations observed in plants’ responses to disease, pathogen infection and wounding, where DE genes are involved in redox regulation. Significant down-regulation of ROPs may contribute to the imbalance of redox regulation, resulting in effects similar to viral invasion, which may be critical for enduring transient exposure to mild chilling stress. Since RmlC-like cupins can recruit genes with functions of nutrient reservoir for adapting to a lower temperature under the CCA condition and ROP genes lead plants to a catabolic, growth-suppressing state under the CS condition, the differences in the regulation of these positive and negative affecting genes may potentially cause the difference in chilling tolerance between the CCA and CS conditions.

### Above-zero chilling resistance after chilling acclimation

By a conventional standard, chilling acclimation is the process by which plants acquire freezing tolerance through exposure to suboptimal, low, nonfreezing temperatures. Plant species vary widely in their abilities of chilling acclimation. The chilling-sensitive species, such as tomato and rice, may be unable to be acclimated to acquire freezing tolerance. However, chilling resistance should not be limited to sub-zero temperatures, especially for tropical and sub-tropical crops. Although being intolerant to freezing, they should be able to tolerate a much lower chilling temperature after chilling acclimation. This increased chilling resistance can allow tropical crops to withstand more severe chilling stresses than before. Unfortunately, little has been done in the past to address the impact of above-zero chilling resistance.

CBF (C-repeat/dehydration-responsive element binding transcription factor) genes are known to be ubiquitous and have conserved structures and functions in a wide range of plant species, including tropical and temperate plants [4],[42],[43] as well as other plants [18],[44]. All four CBF genes in Arabidopsis, in which CBF1, 2, 3 are cold inducible and CBF4 is drought inducible, are arranged in tandem in the Arabidopsis reference genome [45],[46]. In Cassava, 70 CBF homologues can be identified through a sequence search with an e-value less than e^−20^. It is perplexing that despite having such a large number of CBF-like genes, cassava is unable to withstand freezing temperatures. Nevertheless, Cassava can endure chilling temperatures to some extent, particularly after chilling acclimation as shown in our study. It is interesting to observe that none of the 154 miRNAs (93 know and 61 novel), detected by our deep sequencing data, targeted any of CBF genes. Furthermore, these CBF genes, as well as the homologues of CBF constitutive activator ICE [47],[48], had insignificant expression variation in chilling treatments partly due to their low expression levels. Those results are consistent with the results from an early Cassava chilling study [22], suggesting that the CBF regulon in Cassava may be different from its counterparts in *Arabidopsis*. On the other hand, not being chilling responsive at the transcriptional level did not mean that the CBFs were not responsive to chilling stress because CBFs may also be subjected to ubiquitination and sumoylation regulation at the translational and posttranslational levels, as shown in *Arabidopsis*[49]–[52].

The overexpression of SsDREB1 in *Arabidopsis* could result in chilling-regulated gene expression and freezing tolerance [53]. Conversely, constitutive overexpression of either LeCBF1 or AtCBF3 in transgenic tomato plants does not provide a detectable freezing tolerance [54]. The regulatory functions of the CBF regulon may be similar, but the whole CBF chilling-response pathway could be different between the tropical and temperate plants and the other plants. One clue to understand this difference may come from the upstream regulation components. There are probably different upstream motifs of the CBF regulon and some other transacting components in the regulatory elements of the CBF genes. Another possible reason can be attributed to the genes downstream of the CBF regulon, which lack functional CRT/DRE elements in the promoters of genes that are required in order to affect chilling acclimation response or freezing tolerance. As Zhang et al. [54] suggest that differences among plant species with respect to the DREB/CBF regulon could be due to limited DRE/CRT-like elements in the genomes of chilling sensitive species, which may be the case for rice. The upstream regulation elements, such as cis-regulatory motifs and trans-factors, should be further examined in the DE gene identified in our study.

McKhann et al. [55] analyze the CBF genes and their promoters diversity in the Arabidopsis versailles core collection containing 48 accessions that maximize the naturally occurring genetic diversity, and found polymorphisms in the CBF genes along with differences in CBF and COR gene expression. Although there may be more CBF and COR gene expressed in tolerant accessions, there is no simple correlation between CBF regulon and chilling tolerance. By selecting another closely related Solanum species that differs in its chilling acclimation ability, Thomashow and colleagues [2] find that both tested species altered gene expression in response to low temperature to similar degrees with similar kinetics and that both plants have CBF regulons composed of hundreds of genes [18]. These two studies, along with our finding that there was few miRNA or mRNA related to the CBFs’ response to the three designed chilling stress treatments, suggest that a complex network of genes are involved in the chilling acclimation [56]–[59], and that the CBF genes alone cannot explain all phenotypic differences. It suggests that more investigation is required in addition to the CBF regulon, e.g., metabolite networks that are related to low temperature tolerance.

## Conclusions

The results of the transcriptome and microRNAome profiling and the integrated analysis provided three important observations. First, substantial chilling stresses, regardless whether they were severe (4°C) or moderate (14°C), could lead to comparable transcriptome and microRNAome variations and cause perturbation to biological processes of the same or similar functions. Second, dramatic temperature decreases and further temperature decreases after chilling acclimation could result in drastically distinct transcriptome and microRNAome variations even though both stresses reached at the same temperature of 4°C. Third and most importantly, chilling-sensitive Cassava could be accustomed to, through stress acclimation, severe chilling stresses close to freezing temperature (4°C) at least for a short period of time (5 days in the current study). This might be achieved by reversing the expressions of a large number of affected genes, which may potentially alter the regulatory networks, specifically by inducing or overexpressing genes that help preserve nutrients that are critical for stress tolerance.

## Methods

### Plant materials and stress treatments

Stem segments with three nodes of Cassava (*Manihot esculenta* Crantz) cultivars were extracted from 8-month-old plants, and inclined in 3-L pots filled with barren red soil: vermiculite (1:1, v/v), fertilized with Hoagland’s solution [60], to propagate and generate well-balanced seedlings. The solution was renewed with 300 ml quarter-strength solution once a week. After 2 months of planting, the uniform seedlings were subjected to chilling stress treatments. All plants were field grown in Haikou, Hainan, China, during April and June of natural conditions (11 h light, 13 h dark and 25°C during the day and 18°C at night).

Plants of Cassava cultivar SC124 were transferred to normal 24°C illumination incubator for 2 days to set a homogenous starting point, and were treated with three types of chilling stress. 1) Gradual *chilling acclimation* (CA in Additional file 1: Figure S1): temperature was decreased from 24°C to 14°C at the rate of −2°C/h to exert a moderate chilling stress. Temperature was then held constant at 14°C for five days to accommodate chilling acclimation. 2) *Chilling* stress after *chilling acclimation* (CCA): after 5 days of chilling acclimation and growth under 14°C, plants were watered once with Hoagland’s solution, transferred further to 4°C at the gradient of −2°C/h, and cultivated at constant 4°C for another 5 days. 3) *Chilling shock* (CS): normal plants under 24°C grew under 24°C for 5 days in parallel to the CA treatment, and were then subjected to dramatic temperature decline to 4°C at the rate of −4°C/h to ensure the temperature to reach 4°C at the same time as the CCA treatment finished so that these plants had the same age. The effects of the chilling stresses are shown in Additional file 1: Figure S1. In the CA treatment, total RNA was collected at 6 h, 24 h and 5d after temperature reaching 14°C; in both the CCA and CS treatments, total RNA was collected at 6 h, 24 h and 5d after temperature reaching 4°C. These time points were chosen to account for initial response, secondary response, and functional adaption to chilling stresses. In parallel, plants grown under the *normal condition* (NC) of 24°C were watered once with Hoagland’s solution every 5 days, and total RNA was first collected at 6 h, 24 h and 5d along with total RNA for the CA plants being collected and then at 6 h, 24 h and 5d when total RNA for the CS and CCA plants were collected so as to match samples of chilling treatments.

### RNA isolation, RNA library preparation and NextGen deep sequencing

Three organs/tissues – folded leaf, fully expanded leaf and roots of Cassava cultivar SC124 –were harvested at 6 h, 24 h and 5d for the three chilling treatments of CA, CCA and CS as well as the normal condition as described above. Total RNA was isolated separately for each of the three organs/tissues. The three RNA samples of equal amount for each time point of a treatment were then pooled together for expression profiling. As a result, four mRNA libraries and four small-RNA libraries, corresponding to the conditions of CA, CCA, CS and NC, were constructed.

The four mRNA libraries were sequenced by RNA-seq by Illumina GAII following Illumina RNA-seq protocol. Briefly, total RNAs were isolated, purified and reversely transcribed, the resulting cDNA products were subsequently digested with NlaIII and the 3′-cDNA fragments captured with the oligo(dT) beads, and then ligated to the Illumina GEX NlaIII Adapter 1. The junction of Illumina adapter 1 and CATG site contained the recognition site of MmeI, cutting 17 bp downstream of the recognition site (CATG) to produce tags. After removing 3′fragments with magnetic beads precipitation and MmeI digestion, an Illumina GEX adapter 2 was introduced at the end of tags. The resulting adapter-ligated cDNA tags were subjected to 15 cycles of linear PCR amplification, and then purified and sequenced with the method of sequencing by synthesis (SBS) using Illumina GAII.

The six small-RNA libraries (four for Cassava and two for castor bean) were subjected to small-RNA deep sequencing by Illumina Genome Analyzer (GAIIx). Briefly, total RNA was isolated using RNA plant Reagent kit (TIANGEN, Beijing, China). Small RNAs were enriched by poly-ethylene glycol precipitation, separated on 15% denaturing PAGE, and visualized by SYBR-gold staining. Small RNAs of 16- to 28-nt were gel-purified. Purified small RNAs were ligated to a 5′ adaptor and a 3′ adaptor sequentially, reverse transcription amplified, and sequenced.

All mRNA and small-RNA sequencing data have been deposited into NCBI/GEO under accession # GSE52178.

### mRNA differential expression assay

The RNA plant Reagent kit (TIANGEN, Beijing, China) was used for total RNA isolation. The quantity and quality of extracted total RNAs were detected by 1% agarose gel electrophoresis and spectrophotometer. To assess the expression of an mRNA, the first-strand cDNA products were prepared using Fermentas reverse transcriptase kit (K1611). Real-time PCR was performed following the standard SYBR Premix Ex Taq™ kit (TaKaRa) protocol. The reactions were incubated in 0.1 ml tubes of Rotor-gene 6000 machine. The procedure ended by melt-curve. A negative control (no template) was included for each primer combination.

Gene actin served as a reference for mRNA in each sample, genes were amplified in parallel for 3 replicates. The relative concentration was calculated as 2 powered -△△CT, where △△CT = (△CT sample - △CT control), △CT = CT (target)-CT (reference) in each sample. If the CT value was greater than that of no template control (NTC), the mRNA was considered not expressed. The gene specific primers used in the target validation are provided in Additional file 1: Table S6.

### Experimental miRNA target validation

RNA ligase-mediated rapid amplification of 5′cDNA ends (RLM-RACE) GeneRacer Kit (Invitrogen, USA) was used to validate miRNA-guided mRNA cleavage, which differed with traditional 5′RACE of full-length cDNA by omitting the 5′ phosphates of truncated mRNA removal and the 5′ cap structure of full-length mRNA removal treatments. Briefly, total RNA was extracted with RNAplant regent (TIANGEN, DP407-01), and PolyA RNA was isolated using polyAtract mRNA isolation system III (Promega, USA) to eliminate contaminated non-mRNA. Ligation with a 5′ RNA adapter and a reverse transcription were performed. The resulting cDNA was used as a template for PCR amplification. Two ~100 bp spaced gene specific reverse primers (GSP1 and GSP2) for each target, designed based on the downstream sequence of the miRNA:target binding site at the target gene sequence. Combining with two GeneRacer 5′ forward primers (included in GeneRacer kit) to specifically nest amplify the 3′ cleavage product of the target mRNA. The amplified PCR products were gel purified, cloned and sequenced (Sangon, China). Gene specific primers that we used are provided in Additional file 5: Table S7.

### Processing of RNA-seq and small RNA-seq data

Raw RNA-Seq reads with low quality were firstly discarded. Sequencing adaptors were trimmed using an in-house method that recursively searches for the longest substring of the adaptor appearing within a sequence read. If a raw sequence read did not have a substring of the adaptor longer than 6 nt, it was considered as having no adaptor. The adaptor-trimmed sequences with no ambiguous reads, which were referred to as qualified reads, were then mapped to the cDNA sequences and reference genome of Cassava using Bowtie 0.12.7 [61] allowing no more than one mismatch. For small RNA sequencing data, the raw sequence reads with no 3′ sequencing adaptor, of low quality, or shorter than 17 nt were removed. The adaptor trimming for the small RNA-seq reads was done in the same way as for the mRNA reads. The qualified small RNA reads were mapped to the Cassava genome using Bowtie allowing no more than one mismatch.

### Identification of expressed and differentially expressed mRNA genes

The number of reads mapped onto each mRNA transcript was recorded as a raw read count. A gene was considered as expressed if its CPM (Count Per Million mapped reads) was not less than 10. For genes that had CPM less than 10, we considered the aligned reads as noise and set their expression level to 0. For expressed genes, we normalized the raw read counts using the upper-quartile normalization method [62]. Given two conditions to be compared, a gene was considered differentially expressed if any of the following two criteria was satisfied: (1) the change of normalized counts was no less than 4 folds if the gene was expressed in both conditions, or (2) the gene was not expressed (CPM < 10) under one condition and over-expressed (CPM > 40) under the other condition.

### Identification of miRNA targets and anti-correlated miRNA-mRNA pairs

The TargetFinder program [63] was used for computational prediction of miRNA targets. TargetFinder uses a scoring scheme that charges a penalty of 1 for a mismatch or insertion/deletion and a penalty of 0.5 for a wobble base pairing, and it doubles these penalties for base-pairs in the seed region (the 2-7 nt from the 5′-end) of a miRNA. To reduce false positive targets, we set the threshold for target alignment score to 4 so that only target genes that have alignment score no greater than 4 were retained as putative targets. A pair of miRNA and its putative target was considered anti-correlated if both the miRNA and the target were DE and the target was up or down-regulated in the opposite direction with respect to the miRNA expression change.

### GO function enrichment analysis

The Gene Ontology annotations of Cassava mRNAs were retrieved from Phytozome website v4.1 [64]. The statistical significance of GO annotation enrichment was measured by Fisher’s exact test. For each ontology category, the *p*-value was calculated as the probability under which we would observe at least *k* genes to have a given GO term, *t*, if we randomly selected *m* genes from the given *M* genes in the genome, where *n* genes were associated with term *t*. Given a term and a list of genes, *k* follows the hyper-geometric distribution. We also reported the False Discovery Rate (FDR) based on Benjamini-Hochberg multiple testing correction [65].

### Supporting data

The raw sequencing and processed data from this publication have been deposited into GEO database (http://www.ncbi.nlm.nih.gov/geo/) with accession number GSE52178.

## Abbreviations

CA: Chilling acclimation

CCA: Chilling stress after chilling acclimation

CS: Chilling shock

NC: Normal condition

## Competing interests

The authors declare that they have no competing interests.

## Authors’ contributions

WZ and MP initiated the project and designed the experiments; CZ carried out the chilling stress experiments and gene validation; JX, ZC and KZ processed sequencing data and performed the computational analyses; WZ, CZ, ZC and JX analyzed the results and wrote the paper; XC and WB isolated sequencing total RNAs; YZ, SS and DD performed 5′RACE experiment; XG and BW assayed physiological traits, JZ, HP, WW provided reagents. All authors have read and approved the manuscript for publication.

## Additional files

## Supplementary Material

Additional file 1: Table S1. Statistics of RNA-seq data (raw reads and reads mapped to the reference genome with one mismatches), expressed mRNAs and differentially expressed mRNAs from the normal condition (NC) and three chilling stress conditions (CA, CCA and CS). **Table S2.** Statistics of raw sequence reads from four small-RNA libraries from Cassava (A, B and C) under chilling stress and normal condition. (A) Statistics of raw reads. (B) Statistics of qualified reads mapped to coding and noncoding transcripts/regions with zero mismatches. (C) Statistics of qualified reads mapped to coding and noncoding transcripts/regions with no more than one mismatch. **Table S6.** Protein coding gene specific primers used in qRT-PCR assay. **Figure S1.** Sketch of chilling stress experiments for Cassava transcriptome and microRNAome profiling. **Figure S2.** Four physiological traits evaluated on leaves of Cassava plants among the three chilling stress treatments and the normal control. (A) Number of leaf falling. (B) Chlorophyll content. (C) Malondialdehyde content. (D) Proline content. **Figure S3.** Distributions of length and first nucleotide of sequencing reads in four Cassava small RNA libraries (A) All qualified reads. (B) Reads mappable to the genome with one mismatch. **Figure S4.** The expression heatmap of differentially expressed mRNAs and miRNAs. mRNAs and miRNAs were clustered using hierarchical clustering and are shown in the dendrograms. **Figure S5.** The anti-correlation relationship between 30 DE miRNAs and 48 mRNAs targets which reversed their expression directions from NC to CA and then to CCA. Click here for file

Additional file 2: Table S3. GO enrichment analysis on (A) the DE genes of pairwise comparisons, (B) detailed comparison of enriched pathways between CA vs NC and CS vs NC, and (C) the reversely DE genes from NC to CA and to CCA. Click here for file

Additional file 3: Table S4. A list of 48 DE miRNA-targeting mRNAs which have reversed the direction of fold change from CA vs. NC to CCA vs. CA. Click here for file

Additional file 4: Table S5. (A) Anti-correlated DE miRNAs and DE mRNAs in all six comparisons. The first column indicates the two conditions to be compared. (B) The potential miRNA targeting genes which are associated with the enriched pathways of six comparisons. “011977 m” is short for Cassava4.1_011977m, and the same abbreviation is applied to the other gene symbols. The “number” refers to the ratio of genes regulated by miRNA to the total number of genes in a given process. The green and blue shade emphasizes two genes which are DE in the same enriched pathway in two comparisons, the yellow shade emphasizes the gene which are differentially expressed in the same enriched pathway in four conditions comparison. Additional file 4: Table S6. Protein coding gene specific primers used in qRT-PCR assay. Click here for file

Additional file 5: Table S7. The two rounds of gene-specific primers (GSP1 and GSP2) used in 5′RACE experiment. Target gene “000210 m” is short for Cassava4.1_000210m, and the same abbreviation is applied to the other gene symbols. Click here for file
