# Supplementary material for: Chilling acclimation provides immunity to stress by altering regulatory networks and inducing genes with protective functions in Cassava
Source: BMC Plant Biol. 2014 Aug 5;14:207. doi: 10.1186/s12870-014-0207-5 (PMC4236759; doi:10.1186/s12870-014-0207-5)
Supplement: Additional file 1: Table S1. — Statistics of RNA-seq data (raw reads and reads mapped to the reference genome with one mismatches), expressed mRNAs and differentially expressed mRNAs from the normal condition (NC) and three chilling stress conditions (CA, CCA and CS). Table S2. Statistics of raw sequence reads from four small-RNA libraries from Cassava (A, B and C) under chilling stress and normal condition. (A) Statistics of raw reads. (B) Statistics of qualified reads mapped to coding and noncoding transcripts/regions with zero mismatches. (C) Statistics of qualified reads mapped to coding and noncoding transcripts/regions with no more than one mismatch. Table S6. Protein coding gene specific primers used in qRT-PCR assay. Figure S1. Sketch of chilling stress experiments for Cassava transcriptome and microRNAome profiling. Figure S2. Four physiological traits evaluated on leaves of Cassava plants among the three chilling stress treatments and the normal control. (A) Number of leaf falling. (B) Chlorophyll content. (C) Malondialdehyde content. (D) Proline content. Figure S3. Distributions of length and first nucleotide of sequencing reads in four Cassava small RNA libraries (A) All qualified reads. (B) Reads mappable to the genome with one mismatch. Figure S4. The expression heatmap of differentially expressed mRNAs and miRNAs. mRNAs and miRNAs were clustered using hierarchical clustering and are shown in the dendrograms. Figure S5. The anti-correlation relationship between 30 DE miRNAs and 48 mRNAs targets which reversed their expression directions from NC to CA and then to CCA. [file s12870-014-0207-5-S1.docx]

Supplemental Information for

**Chilling acclimation provides immunity to stress**

**by rewiring regulatory networks and inducing genes**

**with protective functions in cassava**

Changying Zeng, Zheng Chen, Jing Xia, Kevin Zhang, Xin Chen, Yufei Zhou, Weiping Bo, Shun Song, Deli Deng, Xin Guo, Bin Wang, Junfei Zhou, Hai Peng, Wenquan Wang, Ming Peng, and Weixiong Zhang

Table of Contents

[Supplemental Figures 3](#_Toc379555438)

[Supplemental Figure S1 3](#_Toc379555439)

[Supplemental Figure S2 4](#_Toc379555440)

[Supplemental Figure S3 5](#_Toc379555441)

[Supplemental Figure S4 6](#_Toc379555442)

[Supplemental Figure S5 7](#_Toc379555443)

[Supplemental Tables 8](#_Toc379555444)

[Supplemental Table S1 8](#_Toc379555445)

[Supplemental Table S2 9](#_Toc379555446)

[Supplemental Table S3 10](#_Toc379555449)

[Supplemental Table S4 10](#_Toc379555450)

[Supplemental Table S5 10](#_Toc379555451)

[Supplemental Table S6 11](#_Toc379555452)

[Supplemental Table S7 12](#_Toc379555453)

# Supplemental Figures

## Supplemental Figure S1

**Sketch of chilling stress experiments for cassava transcriptome and microRNAome profiling.** Plants grown under the normal condition of 24°C (NC, top right panel) were subjected to a temperature decrease of -2°C/h until reaching 14°C and then cultivated for 5 days, to reach a state of *chilling acclimation* (CA, lower left panel). The chilling acclimated plants were transferred further from 14°C to 4°C by -2°C/h and cultivated for another 5 days, to reach the state of *chilling* stressed after *chilling acclimation* (CCA, low right panel). In contrast, plants grown under the normal condition were first kept under 24°C for 5 days while the CA treatment was exerted on the other plants, and then were subjected to a dramatic temperature drop to 4°C with a gradient of -4°C/h to reach 4°C at the same time as CCA treatment, this treatment is called *chilling shock* (CS, lower middle panel). Supplemental images on leaves are inserted to show the effect of chilling stresses on the given plants.

**
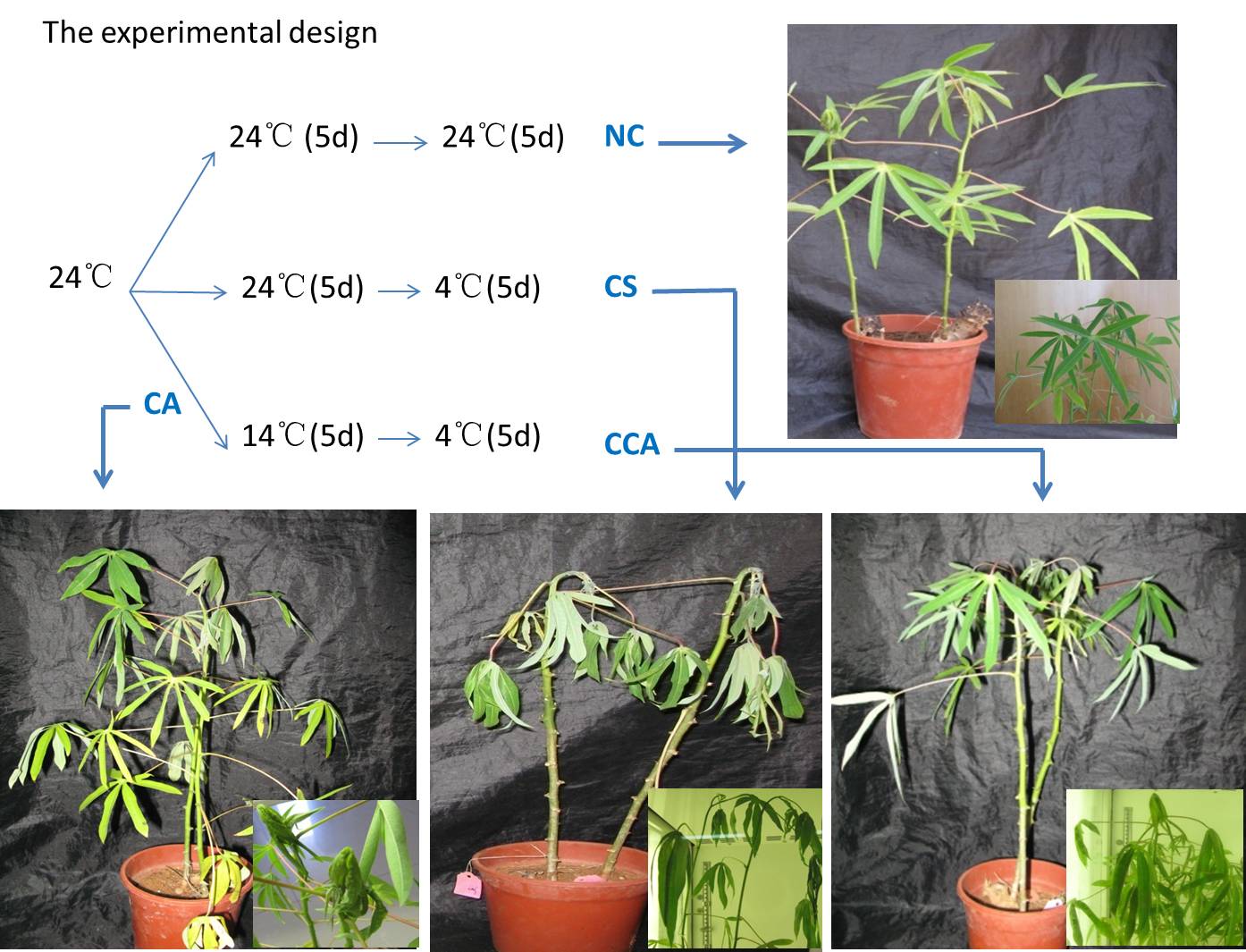
**

## Supplemental Figure S2

**Four physiological traits evaluated on leaves of cassava plants among the three chilling stress treatments and the normal control. (A)** The number of leaf falling. **(B)** Chlorophyll content. **(C)** Malondialdehyde content. **(D)** Proline content.

**
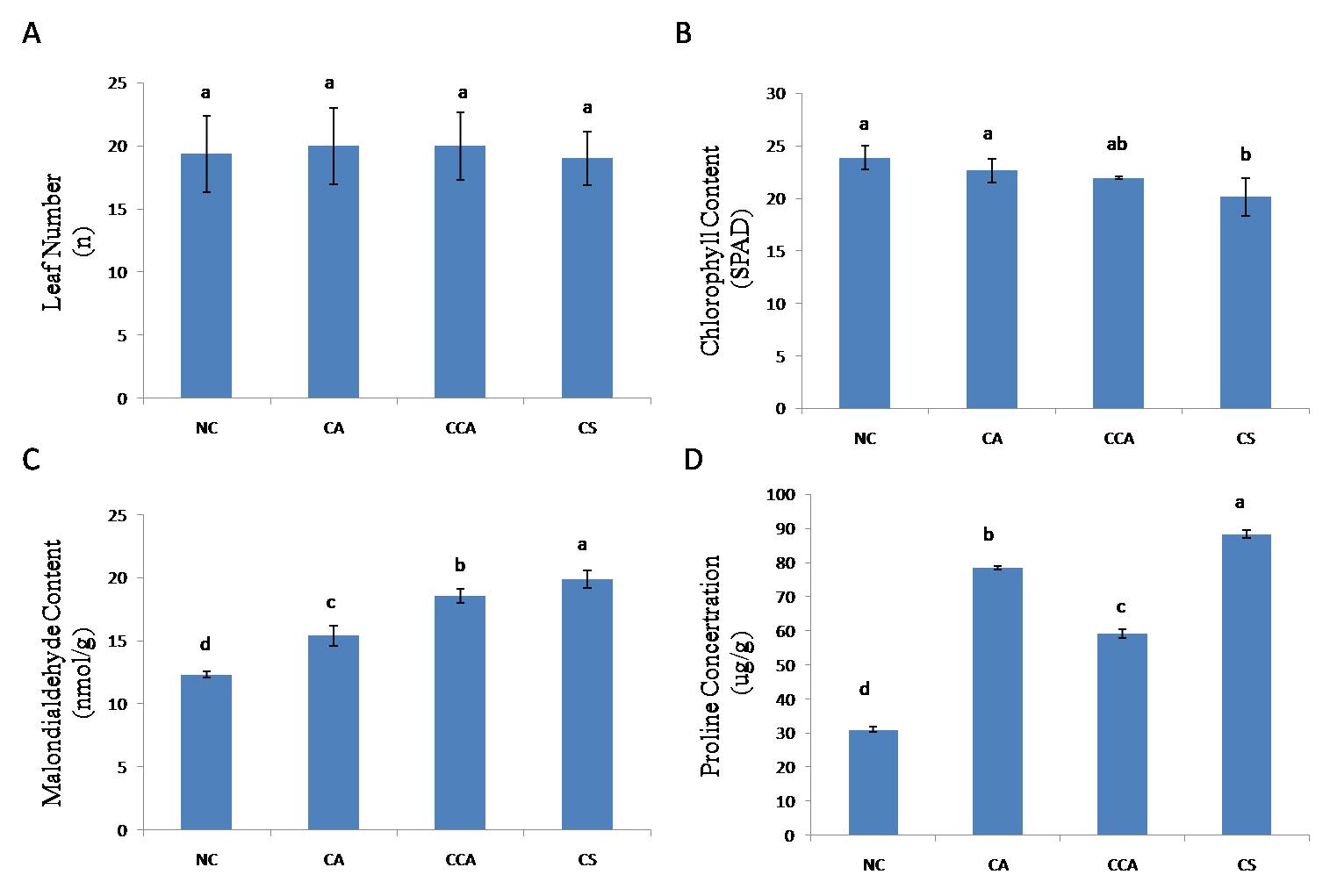
**

## Supplemental Figure S3

**Distributions of length and first nucleotide of sequencing reads in four cassava small RNA libraries**: normal control (NC), chilling acclimation (CA), chilling after chilling acclimation (CCA) and chilling shock (CS).

**(A)** All qualified reads

**(B)** Reads mappable to the genome with one mismatch

## Supplemental Figure S4

**The expression heatmap of differentially expressed mRNAs and miRNAs.** mRNAs and miRNAs were clustered using hierarchical clustering and are shown in the dendrograms.


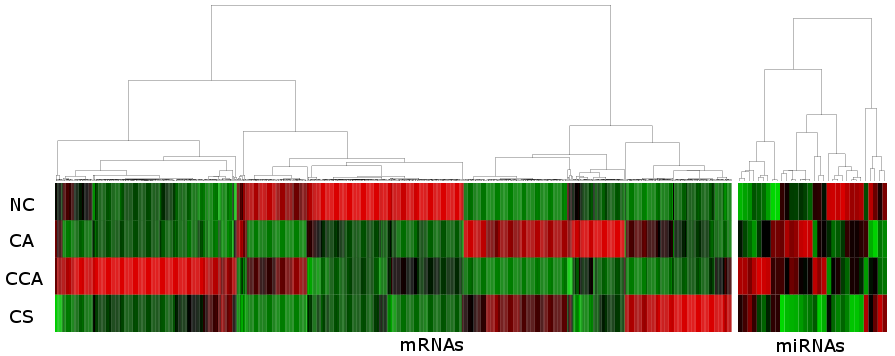


## Supplemental Figure S5

**The anti-correlation relationship between 30 DE miRNAs and 48 mRNAs targets which reversed their expression directions from NC to CA and then to CCA.** The diamonds indicate miRNAs and the circles indicate target mRNAs. An edge between a pair of miRNA and mRNA indicates their anti-correlation relationship. The relative fold-change of miRNAs and mRNA is color-coded, where green denotes down-regulation and red denotes up-regulation.

**
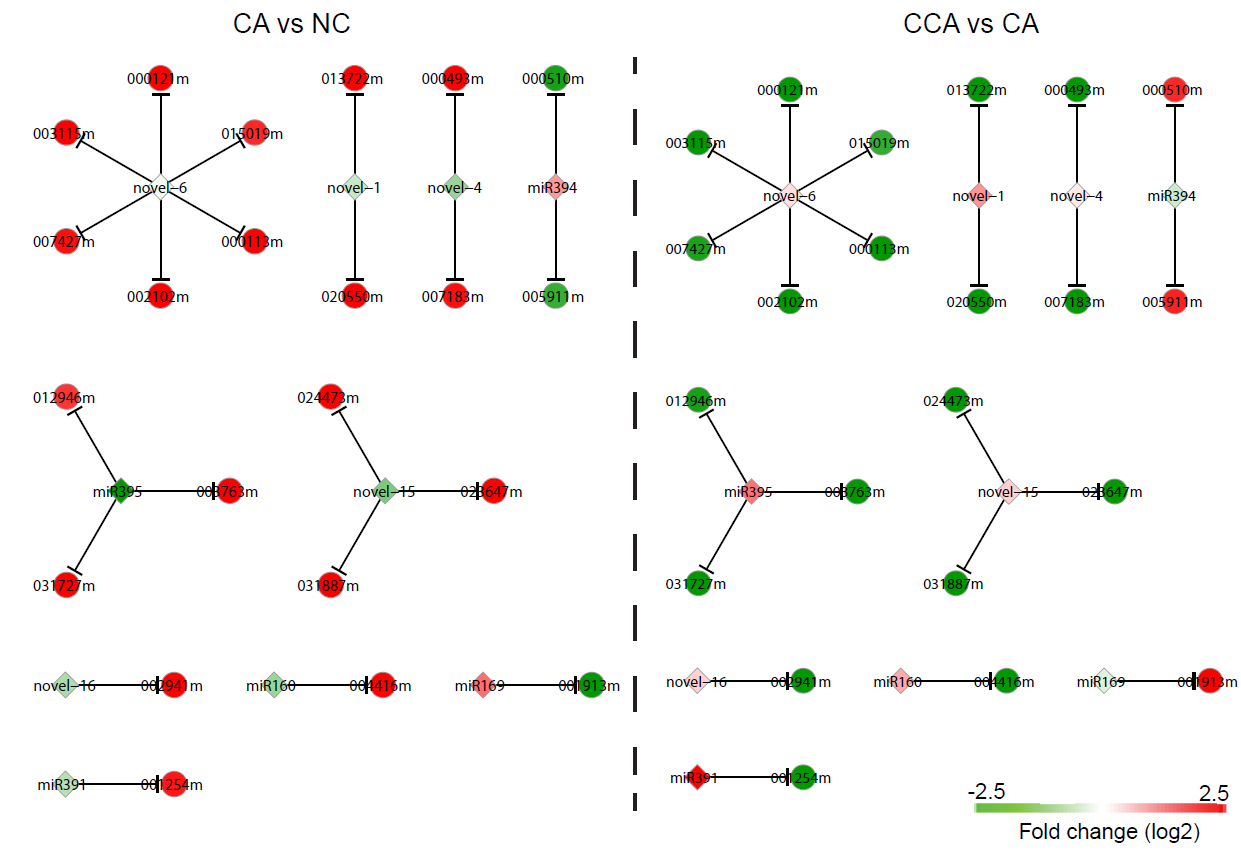
**

# Supplemental Tables

## Supplemental Table S1

**Statistics of RNA-seq data (raw reads and reads mapped to the reference genome with one mismatches), expressed mRNAs and differentially expressed mRNAs from the normal condition (NC) and three chilling stress conditions (CA, CCA and CS).** The percentage of mapped reads was computed based on the number of qualified reads. The percentage of expressed genes was computed based on the number of 34,151 annotated genes in the reference genome.

|  | **NC** | | **CA** | | **CCA** | | **CS** | |
| --- | --- | --- | --- | --- | --- | --- | --- | --- |
| **Category** | **#** | **%** | **#** | **%** | **#** | **%** | **#** | **%** |
| **Raw reads** | 3,825,392 | 100.00 | 13,968,212 | 100.00 | 5,109,859 | 100.00 | 13,828,602 | 100.00 |
| **Qualified reads** | 3,667,388 | 95.87 | 13,532,203 | 96.88 | 4,885,269 | 95.60 | 13,301,727 | 79.86 |
| **Reads mapped to genome** | 3,009,158 | 82.05 | 11,573,485 | 85.53 | 3,689,185 | 75.52 | 10,963,401 | 82.42 |
| **Expressed genes** | 12,689 | 37.16 | 16,023 | 46.92 | 15,144 | 44.34 | 17,026 | 49.85 |
| **DE genes compared to NC** | - | - | 2,855 | 8.36 | 1,083 | 3.17 | 3,298 | 9.66 |

## Supplemental Table S2

**Statistics of raw sequence reads from four small-RNA libraries from cassava (A, B and C) under chilling stress and normal condition.** NC: normal control; CA: chilling acclimation; CCA: chilling stress after chilling acclimation; and CS: chilling shock. Columns headed by # list numbers of raw reads.

**(A)** Statistics of raw reads

| **Condition** | **NC** | | **CA** | | **CCA** | | **CS** | | **Total** | |
| --- | --- | --- | --- | --- | --- | --- | --- | --- | --- | --- |
| **Category** | **#** | **%** | **#** | **%** | **#** | **%** | **#** | **%** | **#** | **%** |
| **Raw reads** | 6670327 | 100.00 | 6391285 | 100.00 | 6616747 | 100.00 | 6015356 | 100.00 | **25693715** | **100.00** |
| **Low quality** | 6765 | 0.10 | 9274 | 0.15 | 12382 | 0.19 | 10893 | 0.18 | **39314** | **0.15** |
| **Short (<17nt)** | 46525 | 0.70 | 63470 | 0.99 | 109238 | 1.65 | 36922 | 0.61 | **256155** | **1.00** |
| **No adaptor** | 489374 | 7.34 | 486456 | 7.61 | 448166 | 6.77 | 505644 | 8.41 | **1929640** | **7.51** |
| **Qualified** | **6127663** | **91.86** | **5832085** | **91.25** | **6046961** | **91.39** | **5461897** | **90.80** | **23468606** | **91.34** |

**(B)** Statistics of qualified reads mapped to coding and noncoding transcripts/regions with zero mismatches

| **Condition** | **NC** | | **CA** | | **CCA** | | **CS** | | **Total** | |
| --- | --- | --- | --- | --- | --- | --- | --- | --- | --- | --- |
| **Category** | **#** | **%** | **#** | **%** | **#** | **%** | **#** | **%** | **#** | **%** |
| **Qualified** | **6127663** | **100.00** | **5832085** | **100.00** | **6046961** | **100.00** | **5461897** | **100.00** | **23468606** | **100.00** |
| **Known miRNAs** | 248255 | 4.05 | 197933 | 3.39 | 233280 | 3.86 | 257574 | 4.72 | **937042** | **3.99** |
| **Novel miRNAs** | 67375 | 1.10 | 67685 | 1.16 | 59833 | 0.99 | 55438 | 1.01 | **250331** | **1.07** |
| **cDNA transcripts** | 403075 | 6.58 | 339017 | 5.81 | 389493 | 6.44 | 409180 | 7.49 | **1540765** | **6.57** |
| **Coding regions (CDS)** | 225230 | 3.68 | 205613 | 3.53 | 217012 | 3.59 | 187490 | 3.43 | **835345** | **3.56** |
| **Mappable to genome** | 3281322 | 53.55 | 3191998 | 54.73 | 3118155 | 51.57 | 2940468 | 53.84 | **12531943** | **53.40** |
| **Unmappable** | 2841858 | 46.38 | 2636091 | 45.20 | 2925144 | 48.37 | 2517705 | 46.10 | **10920798** | **46.53** |

**(C)** Statistics of qualified reads mapped to coding and noncoding transcripts/regions with no more than one mismatch

| **Condition** | **NC** | | **CA** | | **CCA** | | **CS** | | **Total** | |
| --- | --- | --- | --- | --- | --- | --- | --- | --- | --- | --- |
| **Category** | **#** | **%** | **#** | **%** | **#** | **%** | **#** | **%** | **#** | **%** |
| **Qualified** | **6127663** | **100.00** | **5832085** | **100.00** | **6046961** | **100.00** | **5461897** | **100.00** | **23468606** | **100.00** |
| **Known miRNAs** | 395615 | 6.46 | 304271 | 5.22 | 344274 | 5.69 | 404868 | 7.41 | **1449028** | **6.17** |
| **Novel miRNAs** | 79214 | 1.29 | 78923 | 1.35 | 68877 | 1.14 | 63990 | 1.17 | **291004** | **1.24** |
| **cDNA transcripts** | 716292 | 11.69 | 630021 | 10.80 | 690702 | 11.42 | 670438 | 12.27 | **2707453** | **11.54** |
| **Coding regions (CDS)** | 444337 | 7.25 | 415906 | 7.13 | 433700 | 7.17 | 338155 | 6.19 | **1632098** | **6.95** |
| **Mappable to genome** | 4516405 | 73.71 | 4353388 | 74.65 | 4297538 | 71.07 | 4007043 | 73.36 | **17174374** | **73.18** |
| **Unmappable** | 1606938 | 26.22 | 1474880 | 25.29 | 1745849 | 28.87 | 1451316 | 26.57 | **6278983** | **26.75** |

## Supplemental Table S3

**(See Supplemental Table S3.xlsx)**

## Supplemental Table S4

**(See Supplemental Table S4.xlsx)**

## Supplemental Table S5

**(See Supplemental Table S5.xlsx)**

## Supplemental Table S6

**Protein coding gene specific primers used in qRT-PCR assay.**

| Significant enriched biological process | Gene | Forward primer | Reverse primer | PCR product length | Annotation |
| --- | --- | --- | --- | --- | --- |
| Translation | cassava4.1_017802m | TTCTGGGTTTTTGTTTAAGGTCC | AGCAAAGCATTATCCAACAACG | 109 | Ribosomal protein L11 family protein |
|  | cassava4.1_018150m | CAGGCGGTGACTGATCGTAT | ACGTCAAAACGGAAATTGACCA | 181 | ubiquitin 6 |
|  | cassava4.1_019383m | CCTCCAGAGGGATTGAAGGG | ACCTCTGCCATGCTTAGACAC | 291 | Ribosomal protein L31e family protein |
|  | cassava4.1_020116m | GTAGAGGTCATTGTGGGCAGA | TGTGAATAACAACCATCAAACAATG | 169 | Zinc-binding ribosomal protein family protein |
| Nutrient reservoir | cassava4.1_015731m | TGCCTCAGCTTCTGACCCTA | GGGTGAATGTGGGGAGGATT | 288 | RmlC-like cupins superfamily protein |
|  | cassava4.1_029709m | TGACTTTGAAGCAAACGGCG | CAATGCAGCTAGTGCAACCG | 229 | RmlC-like cupins superfamily protein |
| Viral Reproduction | cassava4.1_000174m | CTGAACATGGGCAGGGAGAAT | CCATTGGGCTTCAACGTCTC | 209 |  |
|  | cassava4.1_003690m | GAGCTGGCTCCTCAGAAGTG | ATACTCTGCTTGGGCGCTTT | 201 | ROP interactive partner 3 |

## Supplemental Table S7

**(See Supplemental Table S7.xlsx)**
